# Aberrant DNA Methylation in Keratoacanthoma

**DOI:** 10.1371/journal.pone.0165370

**Published:** 2016-10-27

**Authors:** Yoshimasa Nobeyama, Hidemi Nakagawa

**Affiliations:** Department of Dermatology, The Jikei University School of Medicine, Tokyo, Japan; University of North Carolina at Chapel Hill School of Medicine, UNITED STATES

## Abstract

**Background:**

Keratoacanthoma (KA) is a self-limiting epidermal tumor for which histopathological examination sometimes suggests malignancy. Based on inconsistent clinical views, KA can be regarded as both a benign tumor and a variant of squamous cell carcinoma (SCC). Aberrant DNA methylation frequently occurs in malignant tumors but it scarcely occurs in benign tumors. Whether aberrant methylation occurs in KA has not been previously examined.

**Objective:**

The aim is to elucidate whether aberrant methylation of CpG islands (CGI) containing a high density of cytosine-guanine dinucleotide (CpG) sites occurs in KA.

**Methods:**

Five SCC cell lines, two cultured samples of normal human epidermal keratinocytes (NHEKs), 18 clinical SCC samples, and 21 clinical KA samples were analyzed with Infinium HumanMethylation450 BeadChips, quantitative real-time methylation-specific PCR (RT-MSP) and/or bisulfite sequencing.

**Results:**

Genome-wide analyses of NHEK, KA, and SCC indicated that there was a greater number of aberrantly hypermethylated CGIs in SCC than in KA and there were aberrantly hypermethylated CGIs which are common in both. Among the common hypermethylated CGIs, RT-MSP and bisulfite sequencing targeting CGIs located on *CCDC17*, *PVR*, and *MAP3K11* gene bodies also showed that methylation levels were significantly higher in KA than in normal epidermis. Statistical analyses suggested that the methylation level of CGI located on *PVR* in SCC might be correlated to lymph node metastasis (*P* = 0.013, Mann-Whitney U test) and that the methylation level of CGI in *MAP3K11* in KA might be correlated to age (*P* = 0.031, linear regression analysis).

**Conclusion:**

Aberrant DNA methylation occurs in KA.

## Introduction

Keratoacanthoma (KA) is an epidermal tumor with characteristic clinical and histopathological findings [1]. KA presents with a solitary, pink or flesh-colored, dome-shaped nodule with a central keratin plug [1,2]. A fully developed lesion shows lipping of the edge of the lesion that overlaps the central keratin-filled crater, giving it a symmetrical appearance [1,2]. The tumor lobe consists of large squamous cells with a distinctive pale eosinophilic cytoplasm in the center and basaloid cells in the periphery [1,2]. KA commonly grows rapidly during 1–2 months and spontaneously regress after 3–6 months [1,2]. The tumor develops mainly in chronically sun-exposed areas in the elderly [1,2].

Squamous cell carcinoma (SCC) is a malignant keratinocytic neoplasm in which the component cells show variable squamous differentiation [3]. SCC presents with a shallow ulcer, often with a keratinous crust and elevated, indurated surrounds, or as plaques or nodules [3,4]. The tumor consists of nests, sheets, and strands of squamous epithelial cells derived from the epidermis that extends into the dermis for a variable distance [3]. The cells have abundant eosinophilic cytoplasm and a large, often vesicular, nucleus. The histopathological grade is classified into three types: the well-differentiated type characterized by prominent keratinization and intercellular bridges and limited pleomorphism and mitosis; the moderately differentiated type characterized by less keratinization and prominent pleomorphism and mitosis; and the poorly differentiated type characterized by minimal squamous cell differentiation [5]. The tumor develops mainly in chronically sun-exposed areas in the elderly [3,6]. The surrounding skin usually shows actinic damage [3].

KA and SCC share common characteristics including clinical findings and tumorigenesis. Local tissue destruction can occur during growth and require active treatment. Local recurrence has been reported in up to 8% of KA cases [1,7]. Histopathological examination for KA sometimes shows downgrowth of the squamous epithelium into the dermis with an irregular lower tumor border, perineural and intravenous invasion, and mitoses, as is the case with SCC [1]. Widespread use of *BRAF* inhibitor demonstrates that both SCC and KA can emerge through mitogen-activated protein kinase signal activation [8]. TP53 is expressed in both KA and SCC [9,10]. Based on these common findings, some researchers suggest that it is difficult to distinguish KA from well-differentiated SCC [11], and they regard KA as a variant of SCC [1,12]. According to the World Health Organization classification of tumors, KA is referred to as a well-differentiated SCC (KA type) [1,7].

DNA methylation is the covalent binding of a methyl group to a DNA nucleotide [13]. Methylation of the 5-carbon position of the cytosine in a cytosine-guanine dinucleotide (CpG) plays important roles in mammalian biological function [13]. When a CpG island (CGI) containing a high density of CpG sites on genomic DNA is highly methylated in the 5' region of a gene, the transcription of that gene is suppressed [13,14]. In contrast, DNA methylation in the gene body can promote gene transcription [15,16]. Aberrant DNA methylation frequently occurs in malignant tumors but scarcely occurs in benign tumors. A lot of aberrant DNA methylation in CGI has been elucidated in SCC [17,18,19,20]. However, there is little information with regard to DNA methylation in KA. The aim of the present study is to determine if aberrant DNA methylation occurs in KA.

## Materials and Methods

### Ethics statement

The ethics committee of The Jikei University School of Medicine granted permission for this study. Written informed consent for the use of tissue samples in this research study was obtained from reachable donors or their legal guardians. The ethics committee of The Jikei University School of Medicine waived the requirement for consent from unreachable donors.

### Clinical samples and nucleic acid extraction

SCC cell lines HSC-1 and HSC-5 were provided from the Japanese Collection of Research Bioresources (Tokyo, Japan). SCC cell lines A431 and DJM-1 were provided from the Riken BioResources Center (Tsukuba, Japan). SCC cell line A388 was purchased from the American Type Culture Collection (Manassas, VA). Two normal human epidermal keratinocytes (NHEKs) derived from an adult and a neonate were obtained from ScienCell Research Laboratories (Carlsbad, CA). Eighteen paraffin-embedded SCC samples and 21 paraffin-embedded KA samples were obtained from each patient (Tables 1 and 2). Seven normal skin samples were obtained by shaving the margin of the excised epidermal cysts (Table 3). The TNM classification of SCC was evaluated according to the Union for International Cancer Control TNM Classification of Malignant Tumours (7^th^ edition). The diagnoses of SCC, KA, and normal skin were made histopathologically by at least two experienced board-certified pathologists. To extract DNA from paraffin-embedded samples, the samples were sliced into 4–10 μm–thick sections, deparaffinized, and then dissected with a fine needle. Genomic DNA was extracted by using the QIAamp DNA mini kit (Qiagen, Valencia, CA). Total RNA was isolated by using ISOGEN (Nippon Gene, Tokyo, Japan).

**Table 1:** Patient characteristics of SCC sample donors.

					TNM classification			Methylation level (%)		
ID	Age	Sex	Site	Exposure*	T	N	M	CCDC17	PVR	MAP3K11
1	61	F	nd	nd	2	0	0	89.2	100.0	91.4
2	62	M	Lower ext.	-	2	0	0	66.3	100.0	75.6
3	86	M	Head/Neck	+	1	0	0	8.4	100.0	51.0
4	56	M	Trunk	-	2	0	0	25.8	100.0	75.9
5	98	F	Head/Neck	+	1	0	0	54.4	100.0	82.6
6	80	F	Lower ext.	-	3	0	0	81.0	100.0	55.1
7	96	F	Head/Neck	+	2	0	0	83.5	100.0	43.1
8	71	M	Upper ext.	+	4	0	0	25.9	100.0	80.2
9	77	F	Genitalia	-	2	0	0	26.7	100.0	51.3
10	67	M	Genitalia	-	2	0	0	29.3	100.0	37.4
11	83	M	Head/Neck	+	2	0	0	97.1	95.7	95.4
12	76	M	Genitalia	-	1	0	0	42.9	100.0	70.1
13	69	M	Genitalia	-	2	2	0	90.2	51.2	72.0
14	79	M	Head/Neck	+	2	1	0	68.4	62.7	95.7
15	78	F	Trunk	-	2	0	0	52.8	90.3	41.1
16	83	F	Lower ext.	-	2	0	0	36.8	91.2	51.7
17	90	F	Lower ext.	-	1	0	0	92.0	72.9	85.6
18	79	M	Lower ext.	-	2	0	0	79.7	92.8	92.7

nd, no data.

* Chronic sun exposure.

**Table 2:** Patient characteristics of KA sample donors.

					Methylation level (%)		
ID	Age	Sex	Site	Exposure*	CCDC17	PVR	MAP3K11
19	46	M	Head/Neck	+	na	na	na
20	38	F	Head/Neck	+	na	na	na
21	67	F	Head/Neck	+	29.9	36.7	78.1
22	81	M	Upper ext.	+	35.8	82.1	81.2
23	42	M	Head/Neck	+	29.7	72.8	45.6
24	65	M	Head/Neck	+	68.2	74.8	91.7
25	79	M	Head/Neck	+	65.7	65.1	74.8
26	61	M	Head/Neck	+	49.0	78.1	83.8
27	81	F	Head/Neck	+	83.3	100.0	92.4
28	69	M	Upper ext.	+	79.8	100.0	100.0
29	77	M	Upper ext.	+	60.0	100.0	88.3
30	38	M	nd	nd	51.1	70.4	72.8
31	63	F	Head/Neck	+	41.5	100.0	65.4
32	60	M	Head/Neck	+	56.0	100.0	100.0
33	58	M	Head/Neck	+	54.5	81.9	84.8
34	76	M	Lower ext.	+	41.9	84.9	75.8
35	52	M	Head/Neck	+	36.9	70.0	65.8
36	77	F	Head/Neck	+	70.3	83.8	73.2
37	76	F	Head/Neck	+	78.1	80.2	88
38	60	M	Head/Neck	+	86.1	82.7	88.3
39	50	M	Head/Neck	+	100.0	89.6	25.1

na, not applicable.

* Chronic sun exposure.

**Table 3:** Patient characteristics for normal epidermis sample donors.

					Methylation level (%)		
ID	Age	Sex	Site	Exposure*	CCDC17	PVR	MAP3K11
40	68	F	Head/Neck	+	20.1	41.5	29.3
41	60	F	Upper ext.	+	20.8	28.1	11.5
42	63	M	Lower ext.	-	26.7	51.7	32.8
43	55	M	Head/Neck	+	33.6	38.4	47.1
44	34	F	Trunk	-	26.6	33.4	32.7
45	69	M	Upper ext.	+	19.8	29.8	26.0
46	55	M	Trunk	-	25.6	25.2	31.2

* Chronic sun exposure.

### Genome-wide methylation analysis

Infinium HumanMethylation450 BeadChips (Illumina, San Diego, CA) consisting of 485,577 probes with single-nucleotide resolution were used for genome-wide methylation analysis performed by the contract research service of TAKARA (Otsu, Japan). Output data from the GenomeStudio/Methylation module were analyzed with statistical processing software, R (version 2.15.1). The methylation rate was scored on a range of 0.0 (completely unmethylated) to 1.0 (completely methylated). After filtering through the software's algorithms, analyzed regions were classified according to the relative proximity to a gene as follows: TSS1500, sites −1500 to −200 nucleotides from the respective transcriptional start site (TSS); TSS200, sites −200 to 0 nucleotides from the TSS; 5'UTR, sites from the TSS to the translational start site; 1^st^ exon, sites from the TSS to the end of the 1^st^ exon; gene body, sites from the start of the first intron to the translation end site; and 3'UTR, sites from the translation end site to the end of the gene.

### Quantitative real-time methylation-specific PCR (RT-MSP) and bisulfite sequencing

One μg of the *Bam*HI-digested genomic DNA was modified by sodium bisulfite by using the EZ DNA Methylation-Gold Kit (Zymo Research, Irvine, CA) according to the instruction manual and was then dissolved in 40 μl of buffer.

For RT-MSP, 1.0 μl of the sodium bisulfite-treated DNA was amplified with the 7500 Real-Time PCR System (Applied Biosystems, Foster City, CA) by using a mixture of primer sets that were specific to the methylated or unmethylated DNA sequence (M or U set, respectively) and the SYBR Green PCR Master Mix I (Toyobo, Osaka, Japan). The primer sequences are shown in S1 Table. The number of molecules of a specific sequence in a sample was measured by comparing its amplification with that of standard samples containing 10^1^ to 10^8^ copies of the molecule. The methylation level was defined as the number of methylated DNA molecules divided by the total number of methylated and unmethylated DNA molecules. DNA methylated with *Sss*I methylase (New England Biolabs, Beverly, MA) and DNA amplified with the GenomiPhi DNA amplification kit (GE Healthcare Bioscience, Little Chalfont, UK) were used as methylated and unmethylated DNA controls, respectively, under specific amplification conditions.

For bisulfite sequencing, 1.0 μl of the sodium bisulfite-treated DNA was used for PCR with primers common to methylated and unmethylated DNA sequences. The primer set sequences are shown in S1 Table. The PCR products were cloned into a cloning vector, and 12 clones were cycle-sequenced for each sample.

### Statistical analysis

Statistical analysis was performed by using SPSS version 18 (SPSS Japan, Tokyo, Japan). Significant differences in data were analyzed by linear regression analysis, the Kruskal-Wallis test followed by the non-parametric multiple comparison test, the Mann-Whitney *U* test, and the log-rank test. *P* values <0.05 were considered statistically significant.

## Results

### Genome-wide analysis detects hypermethylated CGIs in both KA and SCC

To screen CGIs with aberrant DNA methylation in KA, two KA samples (#19, #20), two NHEKs (from an adult and a neonate), and two SCC cell lines (A431 and HSC-5) were examined with the genome-wide methylation analysis platform of HumanMethylation450 BeadChip with 485,577 probes. After filtering through the software algorithms, data from 480,844 probes were suitable for analyses.

To detect CGIs with aberrant DNA methylation, 148,837 CpG sites located within CGIs were regarded as targets of DNA methylation analysis. Among the 148,837 CpG sites, 5,833 CpG sites within 2,794 CGIs showed methylation levels <0.2 in both NHEKs and >0.5 in both SCC cell lines, and 1,977 CpG sites within 1,100 CGIs showed methylation levels <0.2 in both NHEKs and >0.8 in both SCC cell lines, while 323 CpG sites within 237 CGIs showed methylation levels <0.2 in both NHEKs and >0.5 in both KA samples. On the other hand, 158 CpG sites within 129 CGIs showed methylation levels >0.8 in both NHEKs and <0.5 in both SCC cell lines, and 18 CpG sites within 16 CGIs showed methylation levels >0.8 in both NHEKs and <0.2 in both SCC cell lines, while 62 CpG sites within 43 CGIs showed methylation levels <0.2 in both NHEKs and >0.5 in both KA samples (S1 Fig).

To more definitely detect aberrantly methylated CGIs in KA for detailed analysis, we established more stringent standards for selecting the aberrantly methylated CGIs. Among the 148,837 CpG sites, 142 CpG sites within 109 CGIs showed methylation levels <0.2 in both NHEKs, >0.5 in both KA samples and >0.5 in both SCC cell lines, and 75 CpG sites within 61 CGIs showed methylation levels <0.2 in both NHEKs, >0.5 in both KA samples, and >0.8 in both SCC cell lines. No CGIs with a methylation level >0.8 in both NHEKs, <0.5 in both KA samples and <0.5 in both SCC cell lines were detected (S1 Fig).

### RT-MSP shows higher methylation levels of some CGIs in KA and SCC

**Fig 1:**
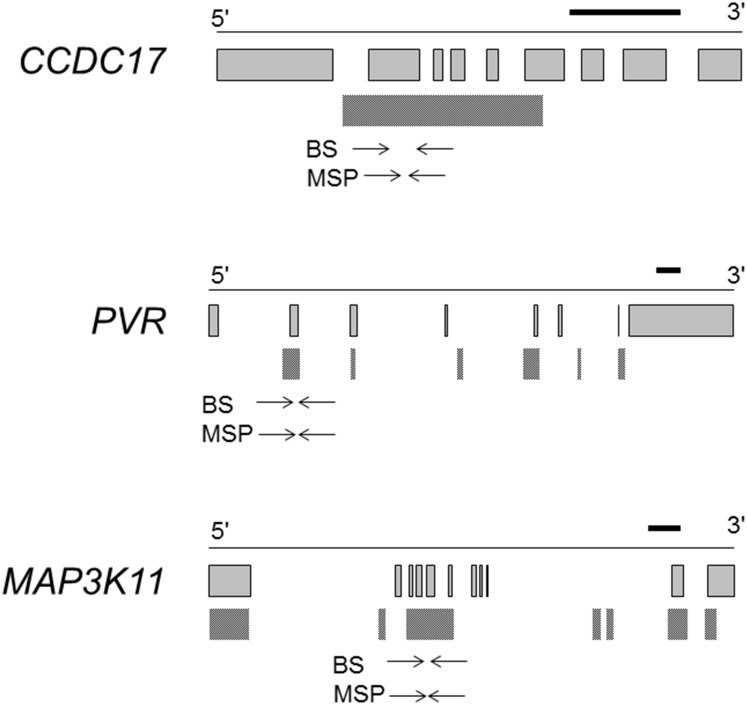
Gene structures of *CCDC17*, *PVR*, and *MAP3K11*. Gray squares and dotted squares indicate exons and CGIs, respectively. The black horizontal bar indicates a length of 1,000 bp. The area between arrows indicates the genomic region analyzed by RT-MSP or bisulfite sequencing.

**Fig 2:**
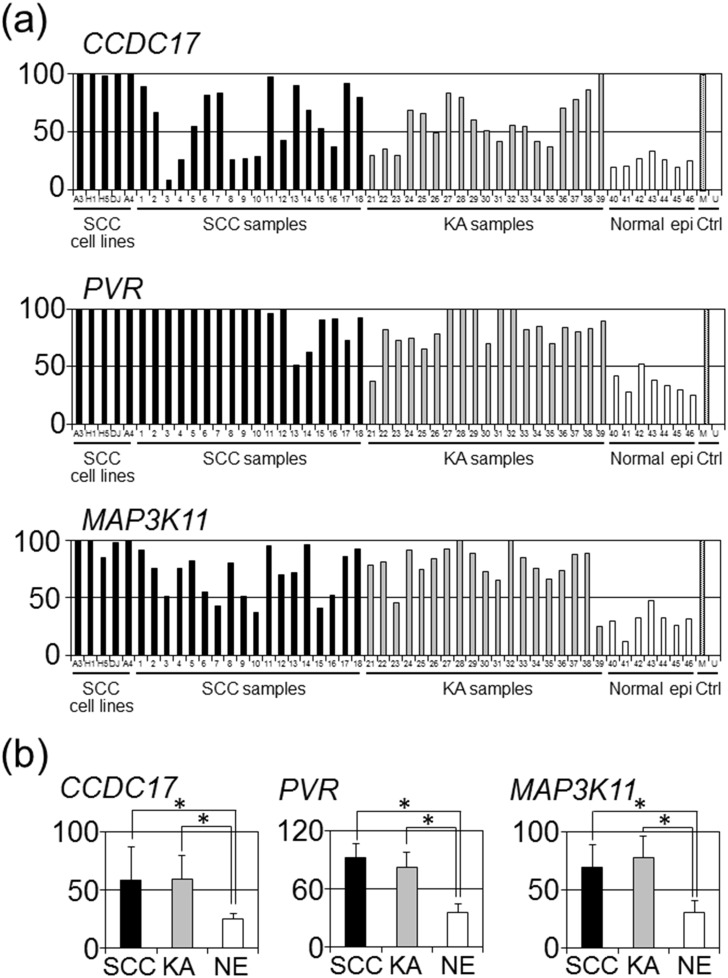
Methylation levels assessed by RT-MSP for CGIs located on *CCDC17*, *PVR*, and *MAP3K11* gene bodies in SCC, KA, and normal epidermis. (a) The methylation level of each sample in SCC cell lines, clinical SCC samples, clinical KA samples, clinical normal epidermis samples, and controls including methylated DNA (M) and unmethylated DNA (U) is indicated as a bar graph. The vertical and horizontal axes indicate the methylation level (%) and sample ID, respectively. (b) Averages and standard deviations of methylation levels in each sample group are indicated as bar graphs. The vertical and horizontal axes indicate the average methylation level (%) with standard deviation and sample ID, respectively. An asterisk represents a significant difference (*P* < 0.05).

Next, we elucidated the methylation level of CGIs detected by genome-wide analysis in additional clinical samples. RT-MSP was performed for CGIs located on the *CCDC17* gene body, *PVR* gene body, and *MAP3K11* gene body, for which RT-MSP primers were successfully developed, in 19 clinical KA samples, 5 SCC cell lines, 18 clinical SCC samples, and 7 clinical normal epidermis samples. Samples used with genome-wide analysis were excluded for the following statistical analyses (Tables 1–3, Figs 1 and 2a). A Kruskal-Wallis test revealed significant differences in methylation levels of each CGI among the three sample groups of SCC, KA, and normal epidermis (*P* = 0.001 in *CCDC17*, *P* <0.001 in *PVR*, *P* <0.001 in *MAP3K11*). Non-parametric multiple comparison tests revealed significant differences between KA and normal epidermis samples (*P* = 0.002 in *CCDC17*, *P* = 0.005 in *PVR*, *P* <0.001 in *MAP3K11*) and no significant difference between KA and SCC samples (*P* = 1.000 in *CCDC17*, *P* = 0.152 in *PVR*, *P* = 0.986 in *MAP3K11*) (Fig 2b).

### Bisulfite sequencing data are compatible with RT-MSP data

**Fig 3:**
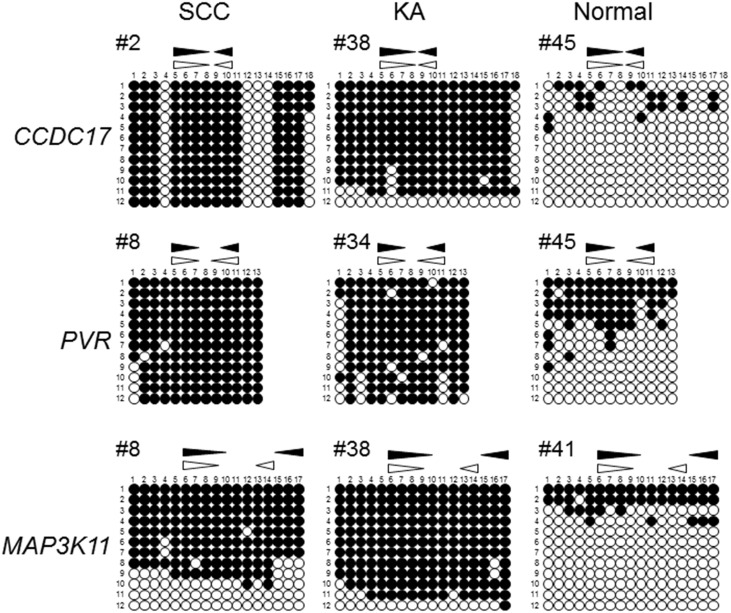
Bisulfite sequencing analysis in CGIs located on *CCDC17*, *PVR*, and *MAP3K11* gene bodies. The results of bisulfite sequencing of the CGIs in representative samples in each sample group are shown. Horizontal rows indicate each CGI located on the *CCDC17*, *PVR*, or *MAP3K11* gene body. The vertical rows indicate each representative sample of SCC, KA, or normal epidermis. The sample ID is the number in the upper left corner of each figure. Closed and open circles indicate methylated and unmethylated CpG sites, respectively. Closed and open triangles indicate the location of the RT-MSP primer sets specific to the methylated and unmethylated DNA sequences, respectively. The vertical and horizontal number rows indicate each clone and CpG site, respectively.

To confirm the data obtained by RT-MSP, bisulfite sequencing was performed for SCC, KA, and normal epidermis samples with representative data for RT-MSP (Fig 3). The examination for CGI located on the *CCDC17* gene body revealed that SCC sample #2 (66.3% methylation level by RT-MSP) and KA sample #38 (86.1%) had densely methylated CpG sites, while normal epidermis sample #45 (19.8%) had sparsely methylated CpG sites. The examination for CGI located on the *PVR* gene body revealed that SCC sample #8 (100% methylation level by RT-MSP) and KA sample #34 (84.9%) had densely methylated CpG sites, while normal epidermis sample #45 (29.8%) had less densely methylated CpG sites. The examination for CGI located on the *MAP3K11* gene body revealed that SCC sample #8 (80.2% methylation level by RT-MSP) and KA sample #38 (88.3%) had densely methylated CpG sites, while normal epidermis sample #41 (11.5%) had sparsely methylated CpG sites. Thus, the bisulfite sequencing data were compatible with the results of RT-MSP.

### Methylation level is possibly related to lymph node metastasis and age

**Fig 4:**
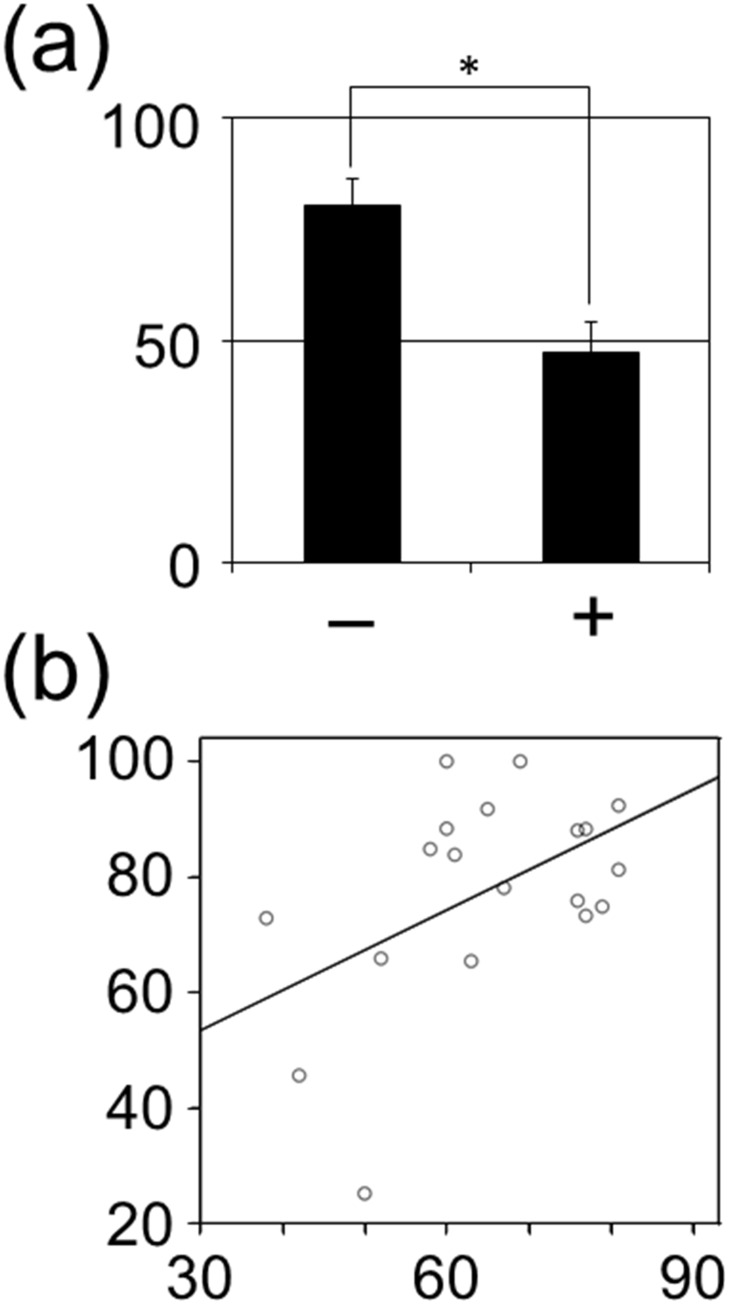
Statistical analyses of methylation levels and clinical parameters. (a) The association between the methylation level of the CGI located on the *PVR* gene body and regional lymph node metastasis in SCC. The vertical and horizontal axes indicate methylation level (%) and regional lymph node metastasis, respectively. Plus (+) and minus (-) indicate presence and absence of regional lymph node metastasis, respectively. An asterisk represents a significant difference (*P* < 0.05). (b) The association between the methylation level of the CGI located on the *MAM3K11* gene body and age in KA. The vertical and horizontal axes indicate methylation level (%) and age (years old), respectively. The methylation level of the CGI located on the *MAP3K11* gene body in KA is correlated to age (*P* = 0.031, linear regression analysis; R^2^ = 0.245).

To investigate the correlation between methylation levels of CGIs located on the *CCDC17* gene body, *PVR* gene body, and *MAP3K11* gene body and clinical parameters of SCC or KA patients, including age, sex, site of occurrence, T-classification, and N-classification, statistical analyses were performed (Tables 4 and 5). The methylation level of the CGI located on the *PVR* gene body in SCC was correlated to regional lymph node metastasis (*P* = 0.013, Mann-Whitney *U* test) (Fig 4a). The methylation level of the CGI located on the *MAP3K11* gene body in KA was correlated to age (*P* = 0.031, linear regression analysis; R^2^ = 0.245) (Fig 4b). These methylation levels were not associated with other clinical parameters.

**Table 4:** *P*-value calculated by each statistical analysis for clinical parameters in SCC samples.

Factors	CCDC17	PVR	MAP3K11	Statistical analysis
Age	0.649	0.893	0.506	Linear regression analysis
Sex	0.360	0.965	0.408	Mann-Whitney U test
Site	0.384	0.881	0.481	kruskal-Wallis test
Exposure	0.568	0.635	0.299	kruskal-Wallis test
T	0.533	0.628	0.930	kruskal-Wallis test
N	0.327	0.013	0.327	Mann-Whitney U test

**Table 5:** *P*-value calculated by each statistical analysis for clinical parameters in KA samples.

Factors	CCDC17	PVR	MAP3K11	Statistical analysis
Age	0.468	0.408	0.031	Linear regression analysis
Sex	0.823	0.687	0.893	Mann-Whitney U test
Site	0.870	0.281	0.452	kruskal-Wallis test
Exposure	0.705	0.481	0.604	kruskal-Wallis test

## Discussion

The present study demonstrated for the first time that aberrant hypermethylation occurs in KA and that aberrant hypermethylation less frequently occurs in KA than in SCC based on the data from genome-wide analysis. Methylation analyses using a genome-wide analysis platform, RT-MSP, and bisulfite sequencing clearly showed aberrant DNA methylation of some CGIs in KA, as is the case with SCC.

Results of a comparison of the genome-wide analysis between *in vivo* KA samples (probably including tumor-surrounding tissues such as fibroblasts, inflammatory cells, and vascular endothelial cells) and *in vitro* NHEKs (including no other types of cells) can be influenced by various types of cells other than KA cells. Therefore, we also compared the *in vitro* SCC cell lines with *in vitro* NHEKs in the analysis of the genome-wide analysis of *in vivo* KA samples. By detecting CGIs with aberrant DNA methylation in both *in vivo* KA samples and *in vitro* SCC samples, the "noise" of surrounding tissues other than KA cells in *in vivo* KA samples can be excluded.

In genome-wide analysis, different cut-off values were also set between SCC samples and KA samples when CpG sites with aberrant methylation were detected in both KA samples and SCC samples. The value to detect hypermethylated CpG sites was set at a methylation level of 0.5 or 0.8 in SCC samples, while it was set at a methylation level of 0.5 in KA samples. The value to detect hypomethylated CpG sites was set at a methylation level of 0.5 or 0.2 in SCC samples, while it was set at a methylation level of 0.5 in KA samples. The *in vivo* clinical KA samples probably included various types of cells, while the *in vitro* SCC cell lines and the *in vitro* NHEKs contained only SCC cells and only normal keratinocytes, respectively. The contamination of non-tumor tissues in KA samples could make the methylation level of the *in vivo* sample closer to that of normal tissue in some CGIs. Therefore, the cut-off value in KA samples should be lower or higher than that in SCC samples to detect CGIs with DNA hypermethylation or hypomethylation, respectively, in KA samples when compared to NHEKs and/or SCC cell lines.

Bisulfite sequencing was performed to confirm the results of RT-MSP. The bisulfite sequencing data were largely consistent with the RT-MSP data. These results suggest that RT-MSP in the present study is a reliable method to assess the methylation levels of CGIs located on *CCDC17*, *PVR*, and *MAP3K11* gene bodies.

Statistical analysis unexpectedly suggested that the methylation level of the CGI located on the *PVR* gene body might be related to regional lymph node metastasis; low methylation levels in the primary lesion might be related to regional lymph node metastasis. To confirm that the methylation level can predict regional lymph node metastasis, a cohort study is needed. We will perform careful follow up on the patients with low methylation levels in the CGI but no lymph node metastasis.

The statistical analysis unexpectedly suggested that the methylation level of the CGI located on the *MAP3K11* gene body might be related to age; a higher methylation level in KA might be related to increased age. The coefficient of determination (R^2^) of 0.245 indicates a weak association between the methylation level and age. Although the data is compatible with the previous report that DNA methylation is affected by aging [21], a larger study is needed to confirm the correlation.

The methylation levels of CGIs on *CCDC17*, *PVR*, and *MAP3K11* gene bodies in KA and SCC samples ranged widely from low levels, which are comparable to levels in normal skin, to high levels of 100%, or almost 100%. These wide ranges could be caused by considerable contamination with tissues surrounding the tumor that reduced the methylation levels in *in vivo* KA or SCC samples. Alternatively, the CGIs might be less methylated in some KAs or SCCs, suggesting that the hypermethylation of CGIs is not essential for tumorigenesis in KA and SCC.

Previous literature describes the functions of proteins encoded by *PVR* and *MAP3K11*, although the function of the protein encoded by *CCDC17* has not been studied in detail. *PVR* encodes CD155 of a transmembrane glycoprotein belonging to the immunoglobulin superfamily. Soluble isoforms of CD155 are increased in various cancers [22]. CD155 plays a key role in cell motility during tumor cell invasion and migration [23]. These findings might be compatible with our data of *PVR* gene body methylation perhaps leading to gene transcription promotion in KA and SCC. On the other hand, Knackmuss et al. reported that MAP3K11 protein might function as an important tumor suppressor neutralized by oncomiR-125b in B-cell leukemia [24], although the function of MAP3K11 in epidermal tumors is unknown.

A limitation of this study is that the results of other cut-off values in the genome-wide analysis were not studied. The analyses might be enormous when various cut-off values are challenged. Since the main aim of the present study is to determine if aberrant DNA methylation occurs in KA, we selected only a few cut-off values in each genome-wide analysis of KA samples, SCC cell lines, or NHEKs, although the values may be subjective.

## Conclusions

The present study demonstrated that aberrant DNA methylation occurs in KA.

## Supporting Information

S1 Fig Number of CpG sites with aberrant methylation in KA and SCC detected by the genome-wide analysis. Vertical axis indicates number of CpG sites. Horizontal axis indicates samples of NHEK (N), KA (K) and SCC (S) with cut-off values. (a) Bar chart indicating number of hypermethylated CGIs compared to NHEKs. (b) Bar chart indicating number of hypomethylated CGIs compared to NHEKs.
(TIF)

S1 Table Primers for MSP and bisulfite sequencing. (DOCX)
