# Supplementary figures and images for: Aberrant DNA Methylation in Keratoacanthoma
Source: PLoS One. 2016 Oct 27;11(10):e0165370. doi: 10.1371/journal.pone.0165370 (PMC5082942; doi:10.1371/journal.pone.0165370)

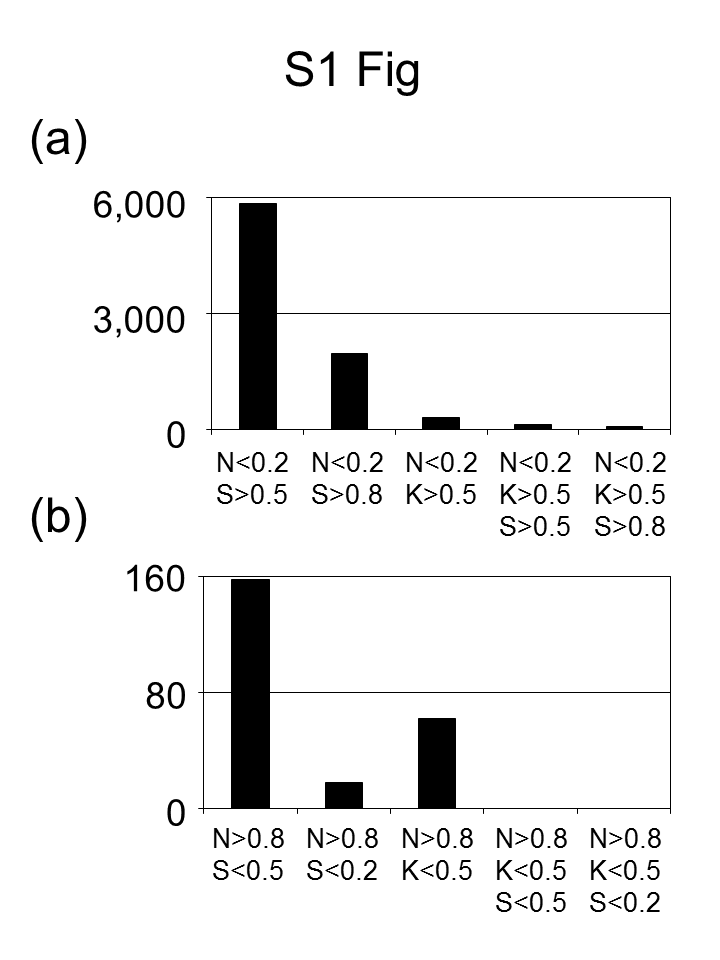

Supplement: S1 Fig — Vertical axis indicates number of CpG sites. Horizontal axis indicates samples of NHEK (N), KA (K) and SCC (S) with cut-off values. (a) Bar chart indicating number of hypermethylated CGIs compared to NHEKs. (b) Bar chart indicating number of hypomethylated CGIs compared to NHEKs. (TIF) [file pone.0165370.s001.tif]
