# Supplementary material for: Aberrant DNA Methylation in Keratoacanthoma
Source: PLoS One. 2016 Oct 27;11(10):e0165370. doi: 10.1371/journal.pone.0165370 (PMC5082942; doi:10.1371/journal.pone.0165370)
Supplement: S1 Table — (DOCX) [file pone.0165370.s002.docx]

**S1a Table.** Primers for MSP

Gene M/U Sequence AT (°C)

Forward Reverse

*CCDC17* M CGGGATATTTCGTTTCGC CCTCAAAACTAAACCGACG 60

*CCDC17* U GGTGGGATATTTTGTTTTGT TCCCTCAAAACTAAACCAACA 61

*PVR* M GTTGAGGATGTTCGGGTTGC CCTACTACCCTACGAAAACG 60

*PVR* U GTTGAGGATGTTTGGGTTGT TACCCTACAAAAACATAACA 58

*MAP3K11* M GTGATCGTTGTTCGCGCGTC CCACCATCGCTATCTCGAACG 60

*MAP3K11* U TGTGATTGTTGTTTGTGT CAAAAACTACTAAACCACA 64

AT, annealing temperature.

**S1b Table.** Primers for bisulfite sequencing

Gene Sequence AT (°C)

Forward Reverse

*CCDC17* GGYGAGGGTGAAGGAGATG CCCCAACACCTCTAAAACT 57

*PVR* GGTTGGAATTYGTGGTAGTTAG CCCTACTCACCAAACACT 57

*MAP3K11* GGGATTGGTGTTAGGTTGAT ACACCTCTAACTCCCACT 57

Y indicates cytosine or guanine. AT, annealing temperature.
